# Transient Apical Breakdown: Incidence, Pathogenesis, and Healing

**DOI:** 10.1111/edt.13002

**Published:** 2024-10-24

**Authors:** Mitsuhiro Tsukiboshi, Yuli Berlin‐Broner, Liran Levin

**Affiliations:** ^1^ Private Practice Aichi Japan; ^2^ University of Alberta Edmonton, Alberta Canada; ^3^ College of Dentistry, University of Saskatchewan Saskatoon Saskatchewan Canada

**Keywords:** apical periodontitis, avulsion, concussion, dental trauma, intrusion, luxation, resorption

## Abstract

**Background/Aims:**

Transient apical breakdown (TAB) is a phenomenon that indicates temporary apical periodontal destruction and root resorption after tooth luxation injuries, followed by the healing process of the dental pulp. Andreasen in 1986, reported that TAB was seen in approximately 4.2% of all luxation injuries. However, there have been no reports thereafter on the types and frequency of the luxation traumatic injuries in which TAB occurs. Therefore, this retrospective study was aimed to assess the incidence and pathogenesis of dental trauma‐induced TAB and to suggest a possible mechanism of subsequent healing based on a series of cases.

**Methods:**

Data analysis included mature teeth (*n* = 56) of 49 patients aged 9–30 years who presented in a private dental office over a period of 10 years (2012–2022) to investigate the incidence and healing sequala of TAB.

**Results:**

TAB was observed in 43.8% of subluxation, 62.5% of extrusive luxation, and 75% of lateral luxation injuries. The average age of patients who developed TAB was 14.5 years, ranging from 9 to 28 years old.

**Conclusions:**

TAB can be expected in many cases of luxation injuries with minimal dislocation. Therefore, mild injuries (subluxation, extrusion, and lateral luxation), may exhibit spontaneous healing, recovery of dark discoloration of the crown, disappearance of a periapical radiolucent lesion and return to normal response to EPT as long as 12 months after the traumatic injury. Thus, a decision to perform endodontic treatment in these cases might be postponed until clear evidence for an infection exists.

## 1 Introduction

The term “transient apical breakdown (TAB)” was originally described by Andreasen FM in 1986 [1]. TAB was referred to the form of either a pronounced radiolucency which appeared spontaneously sometime after injury, or a persistent expansion of the periodontal ligament space (PDL) over an extended period after the injury. At later follow‐ups it had either returned to normal or was accompanied by surface resorption or pulp canal obliteration without any treatment [1]. TAB was observed in 4.2% (27 of 637) of the permanent luxated teeth, which demonstrated a radiographic change alone, with the most (25 out of 27) demonstrated color or electrometric sensibility‐changes [1]. The injuries were moderate, with the majority being extrusion or lateral luxation, affecting both pulpal and periodontal structures [1].

Even though misdiagnosis of TAB may result in unnecessary endodontic treatment [2], there is only limited research addressing TAB. Currently, there are just a few case reports describing TAB related to traumatic dental injuries [2, 3], and a few describing TAB induced by orthodontic forces [4, 5, 6], and the mechanism remains unknown [1, 2]. Therefore, this retrospective study was aimed to assess the incidence and pathogenesis of dental trauma‐induced TAB, and to suggest a possible mechanism of subsequent healing based on a series of cases.

## 2 Materials and Methods

This paper included patients who presented to a private practice, for assessment and treatment, over a period of 10 years (between 2012 and 2022). All the included patients presented with a chief complaint of dental trauma. The initial assessment for all the teeth included clinical tests (palpation, percussion, mobility, and sensibility [electric pulp tests—EPT]) and radiographic examinations. Only permanent teeth that were diagnosed with subluxation, extrusive luxation, or lateral luxation injuries were included in this study.

Furthermore, only teeth with mature roots, defined as radiographic apical foramen diameter of 0.5 mm or less, were included. All the included cases had periapical radiographs (PA), EPT, and intraoral photographs taken at each visit, including the initial visit, 1, 2, 3, 6 months, 1, 2 years, and thereafter at appropriate times depending on the case. Cone beam computerized tomography (CBCT) was taken at the initial visit, and later when deemed necessary.

The determination of TAB among the cases, was based on the following findings:

Clinical findings:

1. Crown discoloration detected followed by its spontaneous disappearance.
2. EPT change from no response to a positive response.

Radiographic findings:

3 Radiolucent periapical lesion detected, followed by its spontaneous disappearance.
4 Enlarged apical foramen.
5 Apical surface root resorption.
6 Pulp canal obliteration (PCO).

**FIGURE 1:**
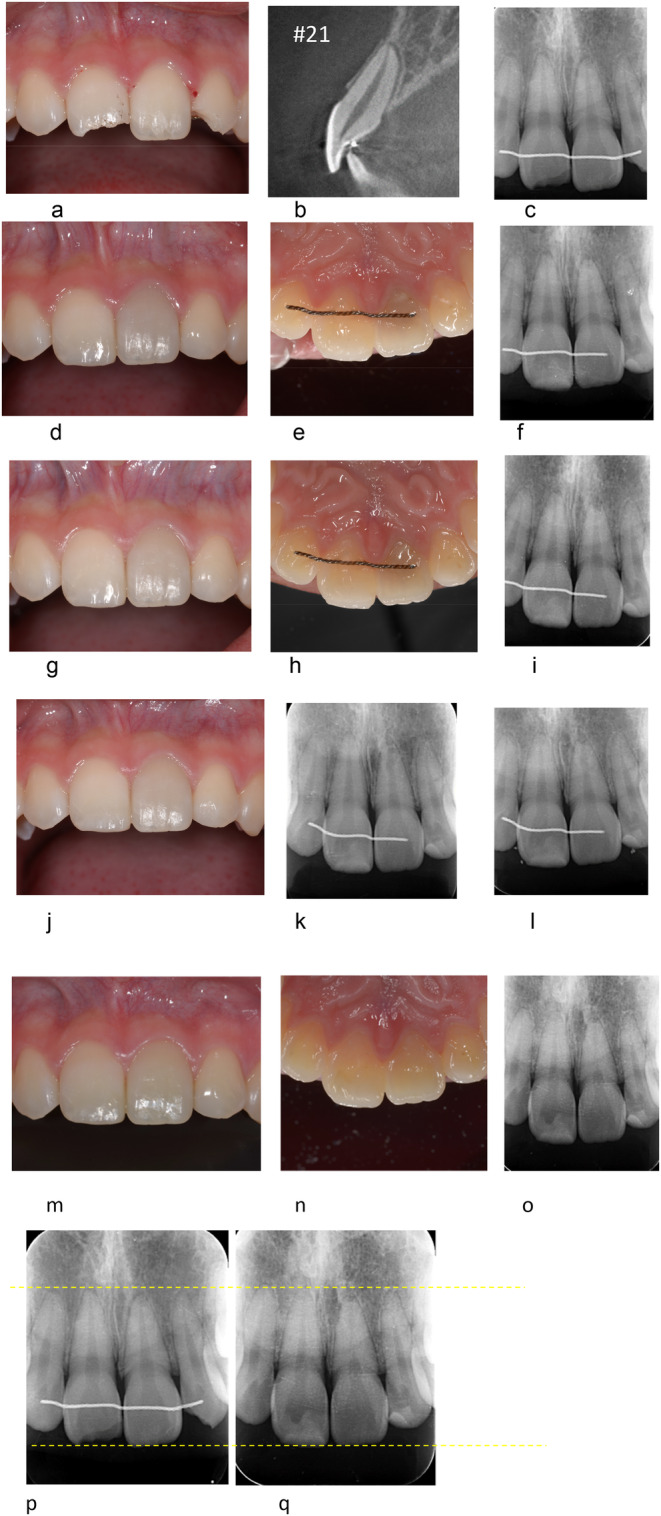
(a–q). TAB after lateral luxation of the left right maxillary central incisor in a 15‐year and 5‐month‐old male who suffered a bicycle accident. (a–c) First visit 1.5 h following the trauma. Clinical photograph is showing anterior maxillary teeth (a). There is a noncomplicated crown fractures on the right maxillary central and the left maxillary lateral incisors. There was a positive response EPT (+) on both teeth. First visit PA radiograph showing no obvious displacement of the teeth (c). CBCT sagittal view of the left maxillary central incisor, showing lateral luxation (b), and it had no response on EPT (−). The anterior teeth had an existing palatal metal wire splint following a past orthodontic treatment before the trauma. (d–f) One‐month follow‐up. Clinical photographs showing apparent brown discoloration of the left maxillary central incisor on the buccal (d) and palatal (e) views. PA radiograph showing no obvious change around the apex (f). There was still no response on EPT (−). (g–i) Four‐month follow‐up. Clinical photographs of the buccal (g) and palatal (h) views showing a slight reduction in the crown discoloration of the left maxillary central incisor. PA radiograph, showing slight apical root resorption of the left maxillary central incisor (i). There was still no response on EPT (−). (j, k) Eleven‐month follow‐up clinical photograph of the buccal view (j) still showing discoloration of the left maxillary central incisor. PA radiograph showing evidence for an enlarged apical foramen on the left maxillary central incisor (k). There was still no response on EPT (−). (l) One‐year and 4‐month follow‐up. PA radiograph showing beginning of PCO in the left maxillary central incisor. At this time sensibility test turned into positive, with EPT (+). (m–o) Three‐year follow‐up. Clinical photographs of the buccal (m) and palatal (n) views of the left maxillary central incisor showing obvious reduction in coronal discoloration. PA radiograph showing progression of PCO in the left maxillary central incisor (o). (p, q) PA radiographs comparing the root length, immediately after trauma, before TAB (p) and 3 years after trauma (q). Resorption and shortening of the root on the left maxillary central incisor are evident (q).

**FIGURE 2:**
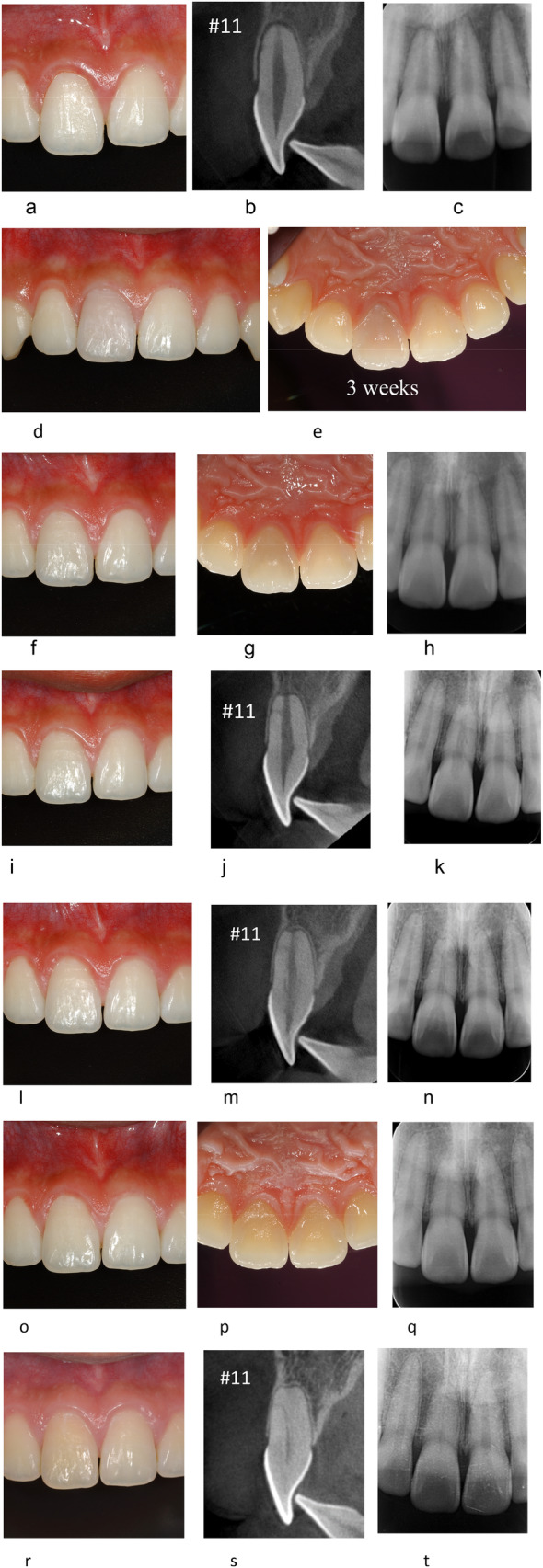
(a–t). TAB after extrusive luxation of the right maxillary central incisor in a 15‐year and 10‐month‐old male. The trauma was caused by a baseball direct hit on the mouth and the visited the clinic 5.5 h following the trauma. (a–) The day of trauma, buccal view photograph showing an extrusive luxation of the right maxillary central incisor (a). CBCT sagittal view of the right maxillary central incisor, showing extrusive luxation of the right maxillary central incisor. The root is fully developed with a closed apex (b). PA radiograph showing an extrusive luxation of the right maxillary central incisor (c). There was no response on EPT (−) on the right maxillary central incisor. (d, e) Three weeks after trauma follow‐up. Buccal view photograph showing slight dark (brown) discoloration of the crown of right maxillary central incisor (d). Palatal view photograph showing slight dark discoloration of the crown of right maxillary central incisor (e). The discoloration is more apparent on the palatal view (e) compared to the buccal view (d). There was no response on EPT (−) on the right maxillary central incisor. (f–h) Six‐month after trauma follow‐up. The right maxillary central incisor was repositioned and splinted for 3 weeks. The discoloration that was evident at 3 weeks after the trauma appears to be reduced as shown on the buccal (f) and palatal (g) views. At this time, the tooth responded to EPT (+). The PA radiograph shows apical root resorption with a widened apical foramen on the right maxillary central incisor (h). (i–k) One‐year after trauma follow‐up. The discoloration is reduced as shown in the clinical photograph of the buccal view of the right maxillary central incisor (i). CBCT (j) and PA (k) images show the apex of the right maxillary central incisor is shortened with a widened apical foramen, and a lateral canal. (l–n) Two years after trauma follow‐up. The crown discoloration is reduced as shown in the clinical photograph of the buccal view of the right maxillary central incisor (l). CBCT (m) and PA (n) radiographs PCO on the right maxillary central incisor. (o–q) Six years and 2 months after trauma. Yellow crown discoloration in the crown is noticeable as shown in the clinical photograph of the buccal (o) and palatal (p) views of the right maxillary central incisor. Further progression of PCO is noticeable on the PA radiograph (q). There are no pathological findings in the periarticular tissues. (r–t) Ten years and 5 months after trauma. Yellow discoloration of the right maxillary central incisor has progressed and is noticeable (r). The pulp canal of the right maxillary central incisor shows progression of PCO (s, t). The pulp canal of the right maxillary central incisor still has a positive response to EPT (+).

**FIGURE 3:**
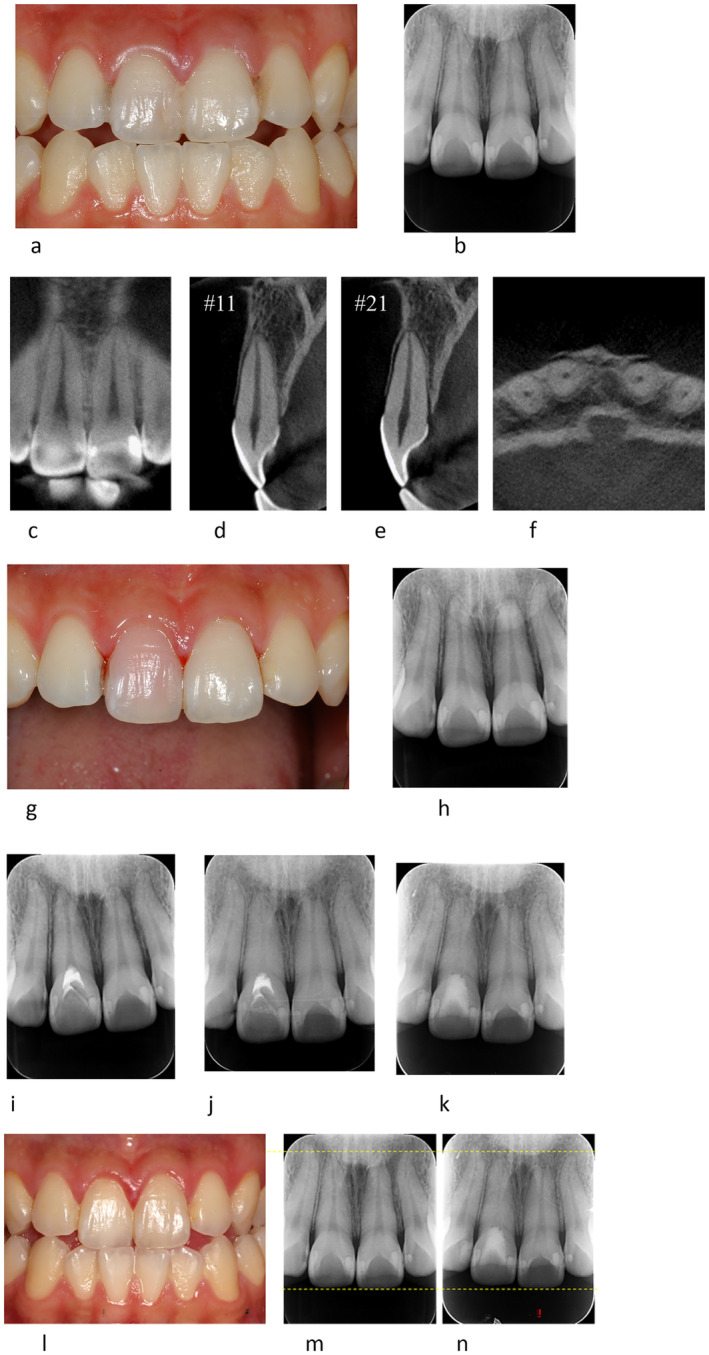
(a–n). TAB, after slight lateral luxation of the right maxillary central incisor in a 20‐year‐old male. The trauma was caused by a friend who bumped their head into the patient's mouth during gym class. (a, b) Clinical photograph (a) and PA (b) radiograph taken 6 days after trauma. Both, the right and left maxillary central incisors had a no response on EPT (−). (c–f) CBCT taken 6 days after the trauma showing both maxillary central incisors are involved in slight lateral luxation, as evident on the sagittal view (d, e) and axial (f) views, with fracture of the alveolar bone as evident on the axial view (f). (g–i) Six weeks after trauma follow‐up. Pink discoloration of the right maxillary central incisor is evident on the clinical photograph of the buccal aspect (g). Thickening of the periodontal ligament space is evident on the PA radiograph of both teeth (h). Both, the right and left maxillary central incisors, still had a no response on EPT (−). Cervical pulpotomy was performed on the right maxillary central incisor. The canal was rinsed with 2% sodium hypochlorite and saline followed by medication with calcium hydroxide (Vitapex, Neo Company, Tokyo, Japan) (i). (j) Ten months after trauma, PCO of the right maxillary central incisor has progressed as evident on the PA radiograph. The calcium hydroxide was replaced with MTA (Bio MTA, imported by Morita, Tokyo, Japan). Root resorption at the apex of left maxillary central incisor is observed. Both maxillary central incisors had a response on EPT (+) at this time. (k, l) One year and 5 months after trauma. PCO of the right maxillary central incisor has progressed as evident on the PA radiograph (k). Both maxillary central incisors still had a positive response on EPT (+). Yellow discoloration of the right maxillary central incisor has been treated by walking bleach performed 6 months earlier (l). (m, n) Comparison of root length between before and after TAB has occurred in the right maxillary central incisor. Six days after trauma (m). One year 5 months after trauma (n). The root of the left maxillary central incisor has been resorbed and shortened, with no changes on the right maxillary central incisor.

Even though almost all the above findings could be found in the TAB cases (Figures 1, 2, 3), there were TAB cases in which crown discoloration or root resorption could not be confirmed. Thus, TAB was also recorded in cases of mature teeth (apical foramen of 0.5 mm or less) with a history of one of the three types of luxation injuries described above, in which sensibility was regained (negative response to EPT changed to a positive response) and PCO occurred, even with no obvious crown discoloration or root resorption.

## 3 Results

The incidence of TAB among the studied patients is summarized in Table 1.

**TABLE 1:** Incidence of transient apical breakdown (TAB) among patients who presented over a period of 10 years (2012–2022), with a chief complaint of dental trauma. Only permanent teeth, with the diagnosis of subluxation, extrusive luxation, or lateral luxation injuries, with mature roots (apical foramen diameter of 0.5 mm or less) were included.

Type of injury	Total number of teeth (%)	Number of teeth with TAB/total number of teeth (%)
Subluxation	32 (57.14%)	14/32 (43.8%)
Extrusive luxation	8 (14.28%)	5/8 (62.5%)
Lateral luxation	16 (28.57%)	12/16 (75.0%)
Total	56 (100%)	31/56 (55.36%)

### 3.1 Demographics

A total of 56 teeth from 49 patients were included and analyzed. Out of them, there were 27 (55.1%) patients with table (22 males, 5 females), and a total of 31 teeth with TAB. The age of the 27 patients with TAB ranged between 9–28 years old (mean 14.5).

### 3.2 Types of Teeth

The 31 teeth that had TAB included left maxillary central incisors (*n* = 16), right maxillary central incisors (*n* = 13), and a right (*n* = 1) and left (*n* = 1) maxillary lateral incisor.

### 3.3 Types of Injury

TAB was detected in 43.8% of subluxation, 62.5% of extrusive luxation, and 75% of lateral luxation cases.

### 3.4 Causes of Injury

Among the 27 patients with TAB, the causes of injury varied between 44% due to sports (*n* = 12), 30% falls (*n* = 8), 11% bicycle accidents (*n* = 3), and 14% due to other reasons (*n* = 4). The most frequent cause of trauma was contact sports, such as basketball and soccer, during which the opponent's elbow or head hit the teeth. More than half (63%) of these injuries occurred inside schools (*n* = 17).

### 3.5 Crown Discoloration

Discoloration of the crown (brown) was observed in 80% (25/31) of TAB cases. Discoloration reached its peak within 1 month, and then showed a tendency to slowly improve within 6–12 months (Figures 1 and 2). The discoloration spontaneously improved in 22 out of 25 cases (17 cases almost completely recovered and 5 cases moderately recovered). In two cases discoloration did not recover spontaneously. There were six TAB cases that showed almost no discoloration. However, in long‐term observation, teeth that showed PCO tended to have a darker shade of the crown compared with teeth that did not. It is noteworthy that improvement of discoloration and EPT returning positive were not related.

**FIGURE 4:**
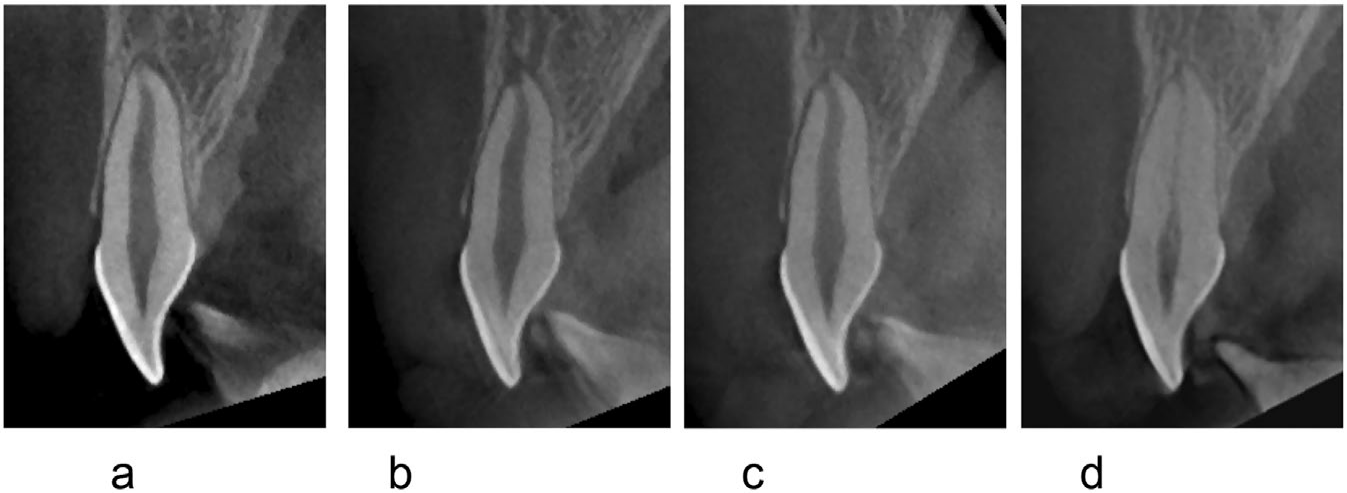
(a–d). TAB after subluxation of the left maxillary central incisor in a 12‐year‐old male due to a bicycle accident. Sagittal views of CBCT scans are presented below. (a) One day after subluxation. The left maxillary central incisor has closed apex and thickening of the apical periodontal space is evident. There was no response on EPT (−). (b) Three months after trauma follow‐up. The left maxillary central incisor shows a small periapical radiolucency, surface root resorption at the apex evident, and there is enlargement of the apical foramen. There was still no response on EPT (−). (c) Six months after trauma follow‐up. The apical foramen is kept wide, with no apical radiolucent lesion evident anymore. There was still no response on EPT (−). (d) Two years after trauma follow‐up. PCO in the left maxillary central incisor is evident. The left maxillary central incisor showed a positive response on EPT (+) since after 1 year and 2 months after trauma.

**FIGURE 5:**
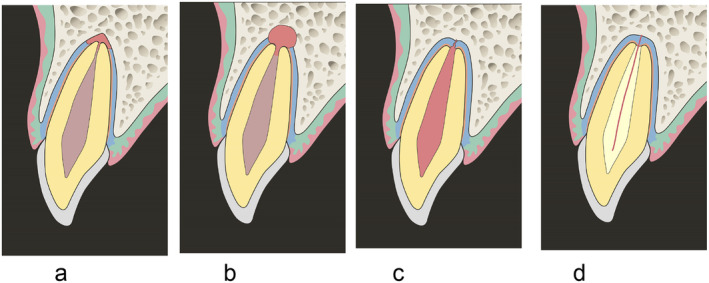
Illustration of a suggested mechanism of TAB in a mature maxillary incisor: (a) Immediately after trauma (subluxation). The pulp is ischemic but not infected. Blood vessels around the apex are raptured and bleeding occurs in a small area. (b) Bleeding induces inflammatory reaction as defense mechanism of the body, which causes osteoclast to disseminate from blood vessels. Osteoclasts cause bone and root resorption, which results in shortening of the root, enlargement of the apical foramen, and a radiolucent periapical lesion or thickening of the apical periodontal ligament. (c) Once the apical foramen becomes wider than 1 mm, revascularization may take place, and the ischemic tissues may be replaced with vital tissues. (d) If cells derived from pulp tissues repopulate the canal, then PCO takes place.

### 3.6 Sensibility (EPT)

EPT became positive within 2–5 months after trauma (*n* = 8), 6–12 months (*n* = 9), 13–18 months (*n* = 6), 19–24 months (*n* = 3), and 25–36 months (*n* = 5).

### 3.7 Pulp Canal Obliteration (PCO)

PCO could be detected radiographically almost simultaneously with EPT, at 6–12 months after the trauma. It took about 2 years for the pulp cavity to be obliterated. Initial obliteration was observed at 6–12 months after trauma (*n* = 14), 13–18 months (*n* = 5), 19–24 months (*n* = 6), 25–36 months (*n* = 6).

## 4 Discussion

TAB is related to the type of injury [7, 8, 9, 10] and is usually caused by moderate injuries to the pulp, such as subluxations, extrusions, and lateral luxation [1, 2, 3, 6], or following orthodontic treatment [1, 2, 3, 4, 5, 6]. Therefore, this study analysis included only these types of injuries. Severe traumatic injuries, such as intrusion and avulsion of mature teeth were not included. These usually involve a permanent vasculature rapture, with no expected return of EPT response. Similarly, concussion is considered a mild traumatic injury, with no vascular rupture at the root apex. Thus, concussion cases were not included in this analysis.

CBCT made it possible to more accurately diagnose the presence or absence of tooth dislocation and determine the type of injury. In the current study, CBCT was acquired in all the trauma cases which had no response to EPT (−). Cases that appeared to be subluxation on a PA radiograph, could be reclassified as lateral luxation on CBCT. This allowed a more accurate classification of the trauma type.

Concussion is considered a mild traumatic injury, with no vascular rupture at the root apex, and no pulp necrosis, thus, positive response to EPT is expected [11]. Therefore, a mature tooth, with no response to EPT (−) after a traumatic injury, and a little tooth dislocation observed on CBCT, was diagnosed as “Subluxation.”

In addition to the type of injury, TAB is related to stage of root development [7, 8, 9, 10]. Therefore, only teeth with mature roots (apical foramen diameter of 0.5 mm or less) were included in the analysis in this study. Teeth with open apices (apical foramen diameter of 1 mm or more), were excluded, since revascularization can be expected without table [7, 12, 13].

Occurrence of TAB in this study was determined by a comprehensive assessment of clinical and radiographic findings. There are several interesting observations from this cohort:

### 4.1 Periapical Radiolucency and Root (Surface) Resorption

The term TAB is derived from the radiographic appearance of an apical radiolucency and apical root resorption [1]. In the current study, it was noticed that when using conventional PA radiographs, TAB could be detected at 1–2 months after trauma. Furthermore, it was noticed that when TAB occurred, apical surface resorption could be evident, and the root length was slightly shortened. In these cases of TAB, the periapical radiolucency disappeared within 6 months.

### 4.2 Enlargement of the Apical Foramen

A characteristic of TAB that was seen in the current study was an enlargement of the apical foramen, which could be detected along with the appearance of an apical radiolucency. Although a PA radiograph can show periapical radiolucency, it may be difficult to detect enlargement of the apical foramen. CBCT enabled detecting the changes in the apical foramen more clearly (Figures 2, and 4). It was inspected that enlargement of the apical foramen in TAB cases could be detected 1–2 months after the trauma, and it remained for 6–12 months, as shown in Figures 2h and 4c. Revascularization is more likely to occur when the diameter of the apical foramen is 1 mm or more [7, 12, 13].

### 4.3 Discoloration of the Crown

In 25 out of 31 (80.64%) of the TAB cases, brown discoloration of the crown, as previously described in the literature [14, 15] has occurred. Discoloration usually peaked within 1 month after trauma and then tended to fade within 6–12 months. The discoloration improved spontaneously in 88% of the cases (22/25). Improvement of discoloration and EPT returning to positive were not related. Teeth with PCO tended to have darker crowns over time when compared to healthy teeth.

### 4.4 Recovery of Tooth Sensibility

In luxation injuries, the apical blood vessels are ruptured, and the pulp suffers ischemic necrosis, which is infection free at first. In this ischemic condition, tooth sensibility is also lost with EPT (−). Eventually, however, when revascularization occurs, and the pulp cavity is replaced by vital tissues, with rapid PCO, and EPT returns to positive [8, 11, 16, 17, 18, 19, 20]. Analysis of the present TAB cases showed that EPT changed from negative to positive starting at 2 months and as late as 25–36 months after the trauma.

### 4.5 Pulp Canal Obliteration

Rapid pulp canal obliteration (PCO) is expected after revascularization [9, 11, 18, 19, 20]. PCO occurs from the peripheral dentinal walls of the pulp of the root canal toward the center, as shown in Figures 1, 2, 3, 4. The degree of obliteration can be variable, from partial to complete obliteration. In the current analysis, TAB cases were followed by PCO. PCO was observed after more than 2.5 years, with one case in which PCO occurred 4.5 years after trauma and EPT became positive at that same time.

### 4.6 Suggested Mechanism of TAB

Based on the clinical observations in this study, the mechanism of TAB might be explained by an early inflammatory reaction with hard tissues resorption and repair process with revascularization and PCO. A suggested mechanism to the histological changes that occur over time during TAB is shown in Figures 4 and 5.

1. Immediately after mild trauma (Figures 4a and 5a): apical vessels are ruptured causing ischemic pulp necrosis (Figure 5a).
2. Day 0–3 months after trauma (Figures 4b and 5b): bleeding and pulp necrosis trigger an inflammatory reaction in the periapical area. During the inflammatory reaction, osteoclasts induce apical bone resorption resulting an apical radiolucent lesion, and a simultaneous root resorption [21].
3. Three to six months after trauma (Figures 4c and 5c): Due to apical inflammatory root resorption, the apical foramen is opened wider than 1 mm, allowing the proliferation of blood vessels into the pulp canal. Pulp revascularization may occur, and the ischemic degenerated pulp is replaced by vital tissues. The resorbed outer root surface is thought to be repaired with new attachment as the inflammation subsides [22, 23].
4. Six months to several years after the injury (Figures 4d and 5d): the pulp canal will show a tendency of rapid calcification (PCO). At the same time, the tooth will respond to EPT (+). This series of healing requires an infection free ischemic pulp.

## 5 Conclusions

TAB can be expected in many cases of luxation injuries with minimal dislocation. Therefore, mild injuries (subluxation, extrusion, and lateral luxation), may exhibit spontaneous healing—recovery of dark discoloration of the crown, disappearance of a periapical radiolucent lesion and return to normal response to EPT as long as 12 months after the traumatic injury. Thus, a decision to perform endodontic treatment in these cases might be postponed until clear evidence for an infection exists.

## Author Contributions

All authors made substantial contributions to the manuscript. This includes conceptualization, methodology, validation, investigation, resources, writing the original draft, reviewing, and editing. All authors have read and approved the final version of the manuscript.

## Ethics Statement

The authors have nothing to report.

## Conflicts of Interest

The authors declare no conflicts of interest.

## Data Availability

The data that support the findings of this study are available from the corresponding author upon reasonable request.
